# Vocational Training in Virtual Environments for People With Neurodevelopmental Disorders: A Systematic Review

**DOI:** 10.3389/fpsyg.2021.627301

**Published:** 2021-07-07

**Authors:** Stefan C. Michalski, Caroline Ellison, Ancret Szpak, Tobias Loetscher

**Affiliations:** UniSA Justice & Society, University of South Australia, Adelaide, SA, Australia

**Keywords:** neurodevelopmental disorder, intellectual disability, Autism, virtual environment, virtual reality, vocational training, work, transfer

## Abstract

People with neurodevelopmental disorders are often considered unsuitable or incapable of working in open employment. When employment is available, tasks are often limited, and opportunities for career development are restricted. Policy and funding constraints leave people with disabilities without an opportunity to develop skills due to the additional time and costs for employers. To overcome these barriers, virtual environments have been proposed as a safe and reliable solution for training. An important prerequisite for a wider uptake of training in virtual environments are demonstrations that the training leads to improved performance in the real world. This is particularly true for people with neurodevelopmental disorders, as transferring learnings from one context to another can be challenging. A systematic review was conducted to assess whether training in virtual environments can be used to improve real-world vocational skills in people with neurodevelopmental disorders. After a systematic search in six databases, eight out of the initially identified 1,806 articles met the inclusion criteria. The findings from these eight studies demonstrate that people with neurodevelopmental disorders can transfer vocational skills from virtual environments to real-world settings. With substantial technological improvements, a surge in accessibility, and improved affordability, there is a need to build upon the promising results identified in this review.

## Introduction

Rates of people with neurodevelopmental disorders in competitive (i.e., open) employment are disproportionately low (Waghorn et al., 2012; Kinoshita et al., 2013; Modini et al., 2016b; Bush and Tasse, 2017). People with neurodevelopmental disorders such as Autism spectrum disorder and intellectual disability encounter considerable challenges in obtaining and sustaining work in open employment. Employment provides numerous benefits, including structure, a source of social support, opportunities to make decisions, income and greater independence (Modini et al., 2016b). Thus, despite frequent challenges such as stigmatising views and a low priority given by employers, it is evident that most still want to work (Kinoshita et al., 2013; Modini et al., 2016a). While open employment is the goal for many, the most common outcome is employment in sheltered workshops or unemployment (Lehman, 1995; Colella and Bruyère, 2011; Cimera et al., 2012; Winsor et al., 2018).

Sheltered employment is a program in which people with disabilities receive training to develop work-related skills and behaviours. The underlying premise is that individuals need preparation before entering open employment; a “train then place” model. The inherent value of sheltered workshops includes tailored support, longstanding social relationships and the opportunity to work. While sheltered workshops may be appropriate for many individuals, the tasks available are often limited, repetitive, and career development opportunities can be restricted. Cimera (2011) suggests only a few individuals transition out of sheltered workshops into open employment. Resource constraints and a lack of innovation are real problems faced by transition educators, making it difficult for people with disabilities to achieve their goals in open employment (Walker et al., 2019). Perhaps if sheltered workshops had access to more flexible, affordable and advanced training tools, they would achieve greater success in transitioning their clients into competitive positions.

Supported employment is an alternative approach where people with disabilities are placed in open employment, often without extensive preparation. The premise is that individuals are placed into competitive positions while receiving intensive on-the-job support; a “place then train” model. Supported employment is not dissimilar to *in-vivo* training, where learners are immediately engaged in a targeted activity in the same location where it is performed in real life. *In-vivo* is generally the preferred method of training as it exposes learners to the work setting and avoids the need to transfer learnings from artificial environments, like in computer-based training. Ideally, people with neurodevelopmental disorders would participate in supported employment all the time, although there are some drawbacks (Choi et al., 2012; Panerai et al., 2018; Lindsay and Lamptey, 2019).

The effectiveness of supported employment is well-established, but challenges remain, which prevent its wide-spread implementation (Marino and Dixon, 2014; Chen et al., 2015). One drawback is concerns surrounding its cost-effectiveness. As trained staff and focused interventions are required, it can become a time and resource-intensive process (Rogers, 2000; Parsons and Cobb, 2011; Marino and Dixon, 2014). Salkever (2013) reviewed the most common supported employment practise (Individual Placement and Support) and found its wide-spread expansion did not support long-term financial gain for employers. In essence, ongoing assistance effectively helps people with disabilities in competitive positions but it is not cost-effective to maintain (Vornholt et al., 2018). Educators and researchers have considered virtual environments to be a cost-effective method to help people in learning environments (Cromby et al., 1996; Bozgeyikli et al., 2014; Smith et al., 2020).

Virtual environments have appealed to educators as a way to bring the real world into the classroom (van Vonderen, 2004; Newbutt et al., 2016b). Immersive and interactive virtual environments may benefit people with disabilities via simulation of training which permits practising as if in real life. Real-world scenarios can be created to provide safe, repeatable and targeted training that focuses on improving the social and practical skills needed in the real world. Various environments and scenarios can be simulated to create realistic interactions for users to practise specific skills and develop confidence before attempting tasks in real-world settings, such as in open employment. Generally, people with intellectual disabilities and Autism spectrum disorder have limited opportunities for skill development in the real world due to concerns surrounding safety and ability (Dempsey and Ford, 2009). Certain life skills make sense for training to occur *in-vivo*; where one will perform the task in real life. Though, for training work-related tasks such as job interviews and the use of hazardous equipment, perhaps there is merit to training in virtual environments (Volkmar et al., 2014).

Virtual environments can be manipulated in ways that take advantage of the learning styles of people with neurodevelopmental disorders (Kandalaft et al., 2013; Knight et al., 2013; Newbutt, 2013; Newbutt et al., 2016a). Features can be enhanced or diminished, objects can be isolated, highlighted or removed, and relationships and associations can be emphasised. The environment can be controlled and manipulated in unique ways that make it particularly interesting for educators (van Vonderen, 2004). Tailored prompts and corrective feedback can be seamlessly integrated into training to reinforce teachings (Kandalaft et al., 2013; Knight et al., 2013; Newbutt, 2013; Newbutt et al., 2016b). Furthermore, visual and auditory cues have been used to help initiate responses and memorise steps in a procedure (Lancioni et al., 1999; Riffel et al., 2005; Mechling, 2007; Sauer et al., 2010; Bailey et al., 2011).

There is encouraging evidence that virtual environments can be used to improve life skills and social skills in people with neurodevelopmental disorders (Standen and Brown, 2005; Ramdoss et al., 2011, 2012). The two most common forms of training using virtual environments for people with disabilities are video-based and computer-based interventions. Video-based interventions involve the learner observing a target skill via video, and then opportunities are provided for the person to imitate the behaviours shown (Ramdoss et al., 2012). There is encouraging evidence to support both video-based interventions (Norman et al., 2001; Rehfeldt et al., 2003; Sturmey, 2003; Mechling et al., 2005; Sigafoos et al., 2005; Rayner et al., 2009; Mechling and O'Brien, 2010; Munandar et al., 2020) and computer-based interventions (Davies et al., 2003; Hutcherson et al., 2004; Hansen and Morgan, 2008; Ayres et al., 2009). However, unlike video-based interventions, computer-based interventions allow the learner to interact via external hardware (e.g., touch screen, keyboard, mouse, joystick) (Mechling and Gast, 2003; Ramdoss et al., 2012). Some view this interaction with the learner as a way of actively involving the participant, which may be beneficial for learning outcomes (Mechling et al., 2005).

The purpose of training in virtual environments is to develop valuable and functional skills to apply in the real world, such as obtaining and sustaining employment. Though, a significant challenge for people with neurodevelopmental disorders that is widely reported in the literature is difficulties in generalising learned skills from one environment to another (Hwang and Hughes, 2000; Rogers, 2000; Parsons and Mitchell, 2002; Westwood, 2009; Ramdoss et al., 2012; Knight et al., 2013). People with neurodevelopmental disorders tend to have difficulty applying learned behaviours in new tasks or contexts, such as in spontaneous situations. Thus, it is unclear how well the skills learned in virtual environments can be applied to real-world settings.

An essential prerequisite for a wider uptake of training in virtual environments are demonstrations that the training leads to improved performance in the real world. That is, the trained skills transfer to the real-world setting. In this paper, *transfer* has been defined as the process by which skills, abilities and knowledge developed through training are applied in a real-world situation or task (Baldwin and Ford, 1988). It is insufficient to assess the effectiveness of virtual environments by only quantifying the extent of improvement in training. This is because the results can almost always be expected to be positive, mainly due to practise effects (Michalski et al., 2019). Measures of real-world performance are therefore needed to determine the added value of training.

The potential benefit of training in virtual environments is clear, but its effectiveness in this population is less certain. Thus, it is necessary to assess virtual environments' effectiveness to improve work-related skills by reviewing articles that measure real-world transfer. This review's primary aim is to synthesise the evidence of virtual environments as a tool to train vocational skills in people with neurodevelopmental disorders.

## Method

### Search Strategy

The Preferred Reporting Items for Systematic Reviews and Meta-Analyses (PRISMA) were followed throughout the review process (Liberati et al., 2009). The review was not pre-registered. A systematic literature search was performed on August 29, 2019. The search was updated on February 8, 2021. The following databases were used: PsycINFO, Ovid Nursing Database, Ovid MEDLINE, Ovid Emcare, and Embase. Psychology, medical and health databases were accessed to cover the scope of this current review. In addition, IEEE Xplore was searched on May 3, 2021. For all mentioned databases, the following search was conducted: [(“virtual reality” OR “virtual environment^*^” OR “augmented reality” OR “mixed reality”) AND (vocation^*^ OR job OR work OR employment OR workplace OR profession^*^ OR occupation) AND (disab^*^ OR mental health OR “intellectual disability” OR “developmental disability” OR “learning disability” OR “brain injury” OR Autism OR retardation OR stroke OR schizophrenia OR bipolar OR depress^*^ OR ADHD OR dyslexia)].

### Inclusion Criteria

Peer-reviewed articles and conference papers published in English were included if they met the inclusion criteria while reviews and meta-analyses were not considered. Each article must have included (a) participants with a neurodevelopmental disorder; (b) a virtual environment; (c) vocational theme; and (d) real-world transfer. Our definition for each factor is detailed in turn.

a) The Diagnostic and Statistical Manual of Mental Disorders, 5th Edition (DSM-5) includes a category of conditions referred to as the “neurodevelopmental disorders.” Studies with at least half of the participants diagnosed with a neurodevelopmental disorder were included.
b) To have been considered a virtual environment in this review it must have included the following components: (i) display or projection of an image; (ii) ability to interact in the environment; (iii) provision of sensory feedback (e.g., visual, auditory or haptic) (Gray, 2017; Michalski et al., 2019).
c) For this current review, a study was considered to have a vocational theme if the study outcome was work/job focused.
d) A study was considered to measure real-world transfer if real-world performance was measured pre- and post-training.

### Article Selection

**Figure 1:**
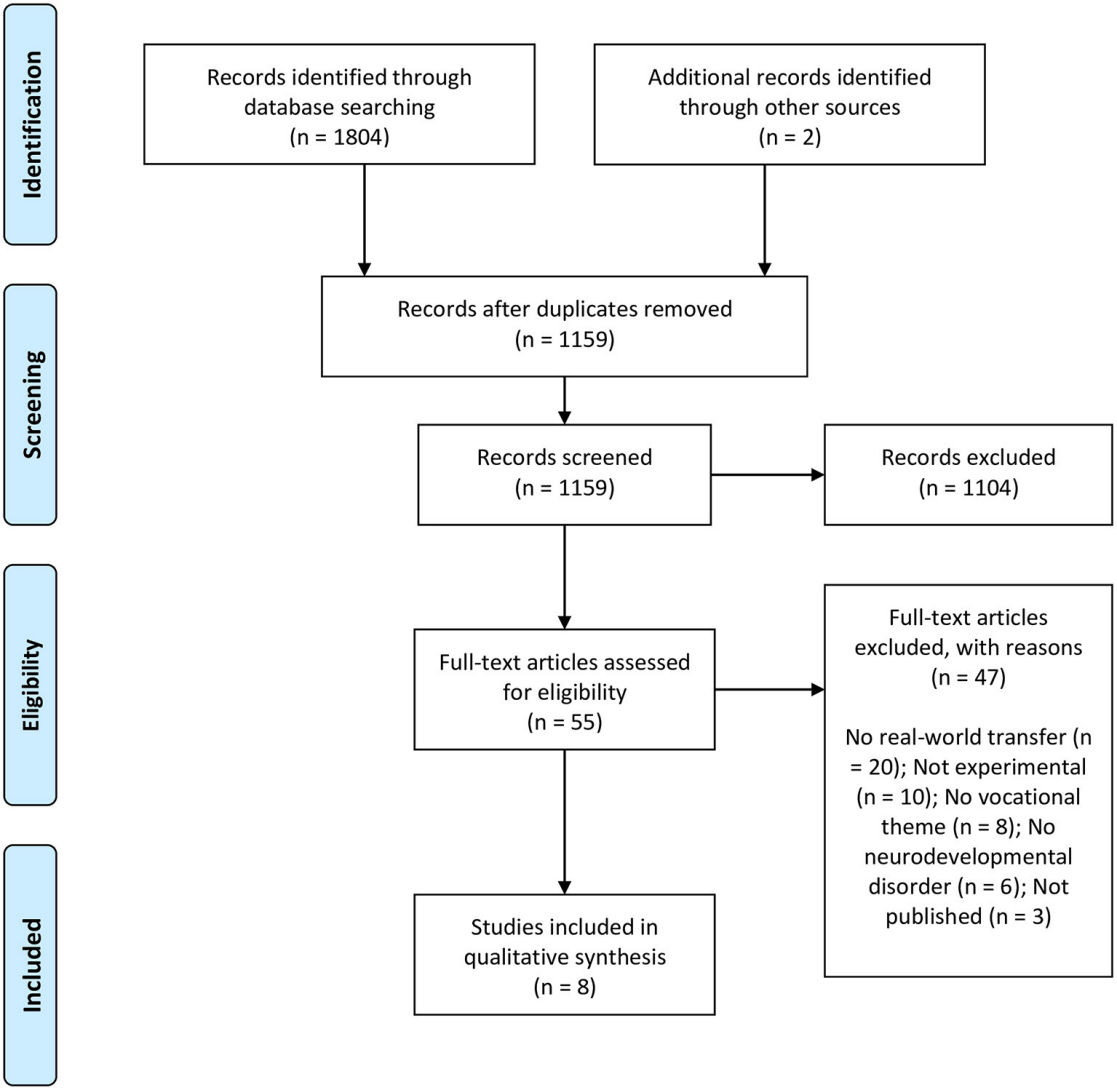
The article selection and screening process using the Preferred Reporting Items for Systematic Reviews and Meta-Analyses (PRISMA) flow diagram (Liberati et al., 2009).

Four reviewers completed the article selection and screening process using Covidence systematic review management software (Covidence, 2020). Titles and abstracts were screened to identify studies that appeared eligible for inclusion. Full texts were sourced and read for articles that appeared eligible or for which eligibility could not be determined. During the full-text reading, articles were included if they met the inclusion criteria. At least two reviewers screened and read each full-text article. Articles were only included when both reviewers agreed. If a conflict arose in any stage during the article selection and screening process, the reviewers resolved the dispute via discussion until a consensus was reached. Reasons for all excluded articles during full-text screening are listed in Figure 1. Additionally, reference lists of included articles were scanned for additional articles.

### Data Extraction

Data extracted from selected studies included information regarding sample, diagnoses, intervention, real-world performance and employment outcomes post-study. Only measures of real-world performance were extracted. Only data from participants with neurodevelopmental disorders were reported. Humm et al. (2014) was excluded as results from participants with Autism could not be separated from participants with other diagnoses.

### Critical Appraisal

A critical appraisal for quasi-experimental studies was used to assess the methodological quality and risk of bias (Tufanaru et al., 2020). Items in the appraisal cover the presence or absence of basic elements of a study, including appropriate statistical analyses, a control group, reliability of measures and group similarity.

Two authors assessed each study. Both authors provided *yes, no, unclear* or *not applicable* responses to each of the nine items in the appraisal. Conflicts were resolved until a consensus was reached. The initial agreement was 90.5% before the two raters resolved conflicts.

*Yes* responses indicate a satisfactory level of quality was reached. All responses were tallied, and *yes* responses were reported as a percentage. Not *applicable* responses (8%) were not included in the analysis. Higher *yes* scores index greater methodological quality with the maximum being 100%. No studies were excluded based on the critical appraisal.

## Results

### Search Results

A total of 1,806 papers were identified through database searching and a snowballing strategy designed to identify additional articles. These publications were imported into EndNote where 647 duplicates were removed. Titles and abstracts were screened for the remaining 1,159 papers. Fifty-five articles appeared eligible for inclusion and were selected for full-text review. Forty-seven articles were excluded with the reasons outlined in Figure 1. Eight studies were included in this systematic review.

### Critical Appraisal

The methodological quality of the included studies varied considerably (37.5–100%), with a mean satisfactory level of 82% across the eight studies.

### Description of Studies

Of the eight articles, two reported multiple assessments resulting in a total of 10 real-world performance assessments. Studies compared training in virtual environments to different control groups: no training (four assessments), real-world training (one assessment), and workbook training (one assessment). Four assessments included pre-post measurements without a control group. Five assessments included participants with intellectual disabilities, three assessments included participants with Autism spectrum disorder, and two assessments included a mixed sample of participants with intellectual disability and Autism spectrum disorder.

Five studies used virtual reality job-interview training programs. These programs require users to engage in conversations with a virtual training agent. While each program is unique, they were similar in that discussion topics varied between sessions, users had freedom in their responses, and the virtual trainers displayed numerous emotions and feedback based on the user's response. Brooks et al. (2002) trained participants to prepare simple meals and identify hazards in a computer-based simulation of a virtual kitchen. Chang et al. (2013) trained meal preparation on a real table with the assistance of a flat-screen display used to provide cues and instructions. Mendozzi et al. (2000) trained simple warehouse tasks, including selecting objects by clicking on them with a mouse and moving them to another location. Mendozzi et al. (2000) also trained workshop skills which included the assembly of a torch, where a flat-screen display was used to provide textual hints to help participants follow the procedure.

### Main Findings

Training in virtual environments significantly improved real-world performance from pre-test to post-test in nine out of 10 real-world assessments. All assessments with no-training controls (four) found training in virtual environments to be significantly more effective than no training. All assessments with real-world training controls (two) found no significant differences in comparison to training in virtual environments. One out of two assessments with a workbook training control found training in virtual environments to be significantly more effective, the other found no significant difference. See Table 1 for a summary of the results.

**Table 1:** Summary of included articles.

**Study**			**Brooks et al. (2002)**	**Burke et al. (2018)**	**Burke et al. (2021)**	**Chang et al. (2013)**	**Mendozzi et al. (2000)**	**Smith et al. (2014)**	**Strickland et al. (2013)**	**Walker et al. (2016)**
Sample	VE group	*n*	24	32 (M25, F7)	150 (M110, F40)	3 (M2, F1)	20 (M14, F6)	16 (M12, F4)	M11	5 (M1, F4)
		Age mean (*SD*)	15–43 (range)	23 (3.12)	21.7 (3.2)	22 (2.6)	25.8 (4.2)	24.9 (6.7)	18.21 (1.03)	20 (1.22)
	Control (specify)	Condition	1) No training 2) Real-world training 3) Workbook	-	-	-	-	No training	No training	-
		*n*	24	-	-	-	-	10 (M8, F2)	M11	-
		Age mean (*SD*)	15–43 (range)	-	-	-	-	23.2 (3.0)	17.66 (1.27)	-
Diagnoses per study sample^*^	Intellectual disability		100%	34%	40%	100%	100%	-	-	100%
	Autism spectrum disorder		-	69%	65%	-	-	100%	100%	-
Intervention	Content/Theme		a) Food b) Hazard awareness	Interview	Interview	Food	a) Warehouse b) Workshop	Interview	Interview	Interview
	Display		Flat screen	Flat screen	Flat screen	Flat screen	Flat screen	Flat screen	Flat screen	Flat screen
	Program		Custom program	Virtual interactive training agent	Virtual interactive training agent	ARCoach	Custom program	PeopleSIM™ technology	VenuGen4	TLE TeachLivE^TM^
	User interaction		Computer-based	Video-based	Video-based	Physical	Computer-based	Computer-based	Video-based	Video-based
	Average time in minutes		-	-	-	-	5,760	532.5 (SD 92.6)	-	-
	Dropouts		-	-	-	-	0%	-	-	-
Real-world performance	Assessment		a) Food preparation b) Hazard identification	Role-play interview	Role-play interview	Food preparation	a) Item retrieval b) Assembly task	Role-play interview	Role-play interview	Role-play interview
	Outcome		Positive	Positive	Positive	Positive	Mixed	Positive	Positive	Positive
	Did VE training significantly improve real-world performance as compared to the no-training control?		a) Yes b) Yes	-	-	-	-	Yes	Yes	-
	Did VE training significantly improve real-world performance as compared to other controls (specified)?		2a) n.s. 2b) n.s. 3a) Yes 3b) n.s.	-	-	-	-	-	-	-
	Did VE training significantly improve real-world performance from pre-test to post-test (within-subjects)?		a) Yes b) Yes	Yes	Yes	Yes	a) Yes b) No	Yes	Yes	Yes
Employment outcomes post-study			-	-	-	-	-	VE group sig. more likely to receive a job at 6 months (Smith et al., 2015b).	-	-

*M, Male; F, Female; VE, virtual environment; sig., significant; n.s., a non-significant difference*.

* *Combined totals may exceed 100% as participants may have had multiple diagnoses*.

## Discussion

This systematic review demonstrates that people with neurodevelopmental disorders can improve vocational skills via training in virtual environments. In nine out of 10 performance assessments, training in virtual environments lead to a significant improvement from pre-test to post-test in real-world settings. Importantly, the findings show that virtual training is more effective than no training. Four assessments with no-training controls found training in virtual environments to be significantly more effective. All available evidence suggests that training in virtual environments can enhance real-world performance in comparison to no training. These findings demonstrate the potential of virtual environments as a complementary tool for training.

No-training controls are essential for determining the added benefit of training but tell us little about its usefulness in comparison to other forms of training. One out of four assessments with an active control group found training in virtual environments to be significantly more effective. It is important to note that non-significant differences were found in the remaining three assessments. Overall, these findings are quite encouraging and suggest that training in virtual environments may be comparable to the outcomes of real-world training.

The findings from this review particularly highlight the effectiveness of conversational virtual human agents to develop job-interview skills. All studies assessing job interview skills found role-play performance improved after training in virtual environments. By practising speech and having the chance to go over their responses, users were able to improve content and delivery skills (Strickland et al., 2013; Walker et al., 2019). Strickland and colleagues (Strickland et al., 2013) found that participants were better able to improve content skills (i.e., verbal skills) in comparison to than delivery skills (i.e., non-verbal skills). However, the studies included were not designed to improve these delivery skills, perhaps due to the limitations in technology at the time. Modern technology may be better equipped to target non-verbal skills in training. For example, posture can be assessed via body sensors, facial expressions can be examined via video recordings, and eye contact can be measured via eye-tracking (Rogers et al., 2018). Thus, while virtual environments in this review were not accommodating for non-verbal skills, future training simulations could target these skills.

Assessments of real-world performance may identify whether participants improved their skills, but this provides no insight to participants' future employment prospects. One of the included studies (Smith et al., 2014) provided follow-up information regarding participants success in finding a job at a 6-month follow up (Smith et al., 2015b). Interestingly, the participants that trained in virtual environments were more likely to be competitively employed in comparison to participants that received no training (Smith et al., 2015b). Similar trends in employment have been found at five and 6-month follow-ups among other clinical populations (e.g., Autism spectrum disorder, bipolar disorder, major depressive disorder, post-traumatic stress disorder, and schizophrenia) (Humm et al., 2014; Smith et al., 2015a,c; Smith et al., 2017). The evidence that training in virtual environments leads to real opportunities is promising, though more studies providing follow-up data are needed to support this finding.

A job interview is a confronting experience and a critical part of obtaining a job in open employment, thus it is essential to teach people the skills needed for an interview. While this is an important step, employers look for individuals which have the necessary skills required for the job (Hall and Wilton, 2011). Three studies investigated skill transfer in applied tasks and found positive results. Mendozzi et al. (2000) assessed skill transfer from virtual environments in an item retrieval and assembly task. Furthermore, Brooks et al. (2002) evaluated the efficacy of using a virtual kitchen for vocational training in food preparation and hazard awareness training scenarios. Finally, Chang et al. (2013) assessed food preparation skills in participants with cognitive impairments. In taking the next steps, more researchers should continue to target skills that people will need on the job. Having an ability to practise skills in safe and repeatable settings may benefit certain training aspects, including procedural learning and adaptation to different work settings.

Training in virtual environments puts the learner in control of the learning situation but having an instructor present might be ideal for providing support in the learning process. In a study by Walker and colleagues (Walker et al., 2016), training job-interview skills in virtual environments was combined with immediate face-to-face coaching. Coaching included a reflection on the participant's training performance and strategies on how to improve future responses. This was designed to promote the generalisation of skills in real-world settings. Ideally, learners' practise with the support of a therapist or caregiver all the time; however, we know one-on-one training is expensive and time-consuming. Perhaps an integration of training in virtual environments with the guidance of an instructor is a practical yet effective approach. If training in virtual environments is to be adopted by organisations, understanding how training should be structured is essential. Information on the dose and frequency of training is therefore critical, yet only a few studies included in this review reported the amount of time spent training.

The available studies training vocational skills in virtual environments have found encouraging results despite using technology that would now be considered outdated. In the eight included studies, three were computer-based, using keyboard, mouse, and joystick interactions. While most studies found positive skill transfer with such interactions, Mendozzi et al. (2000) found mixed results. They found their participants improved in an item retrieval task but did not improve in an item assembly task. These mixed results could be attributed to the technology used. In the study, participants were able to benefit from training in a virtual environment in the item retrieval task, which, by nature, requires fewer complex interactions. However, in the item assembly task of a torch, the physical interactions in training would be considered unrealistic, as using a keyboard and mouse is quite different from assembling objects by hand. This study was published 20 years ago, and technologies are now available to provide more realistic interactions via hand tracking and haptic feedback. It stands to reason that a greater realism in the interactions would improve the learning of practical hands-on skills.

Virtual environments offering a low level of immersion were found to be useful in this review. All studies used flat screen displays in the form of televisions and desktop monitors. This technology would generally be considered to offer a low level of immersion in comparison to what is currently available on the market (e.g., wearable head-mounted displays). Encouragingly, studies in this review reported that sessions were well-attended. As no studies used highly immersive technology, it remains unclear how people with neurodevelopmental disorders may respond in more immersive experiences. Future studies that include immersive virtual environments should report participant dropout rates as this is one indicator of comfort and usability.

Before immersive virtual environments can be encouraged for training people with neurodevelopmental disorders, information regarding adverse effects is critical and must be carefully considered. Self-report measures provide insight on a human level (i.e., how the participant is thinking and feeling), though such findings must be interpreted with caution. There are issues around assessing cybersickness via self-report, and these issues may be confounded in this population. People with neurodevelopmental disorders generally have communication barriers and a tendency to positively self-report (Schwartz and Rabinovitz, 2003; Cummins, 2005). Careful investigation of these responses must be considered to ensure the safe use of virtual reality and reduce the risk of adverse symptoms.

## Conclusion

This review demonstrates that training in virtual environments can be used to improve vocational skills in people with neurodevelopmental disorders. While these results are encouraging, they are only supported by a small evidence base and a very limited range of skills assessed. The authors note 20 articles did not meet the inclusion criteria due to lack of a real-world transfer assessment. There seems a real lack in the translation from prototype to real-world testing. Many studies included in this review used technologies that would be considered outdated today. With substantial technological improvements, a surge in accessibility, and improved affordability, there is a need to build upon the promising results identified in this review. Support organisations may benefit by using virtual environments to boost productivity in the tasks people with disabilities have been commonly assigned to. However, the real advantage is providing people with opportunities to fulfil their goals and ambitions through safe virtual tasks that simulate activities they are typically excluded from in real life.

## Data Availability Statement

The original contributions presented in the study are included in the article/supplementary material, further inquiries can be directed to the corresponding author.

## Author Contributions

SM, CE, AS, and TL conception of the work, article selection, and screening. SM wrote the manuscript. All authors revised the work critically for intellectual content and have read and approved the manuscript.

## Conflict of Interest

The authors declare that the research was conducted in the absence of any commercial or financial relationships that could be construed as a potential conflict of interest.
